# A Comparison of Nine Machine Learning Mutagenicity Models and Their Application for Predicting Pyrrolizidine Alkaloids

**DOI:** 10.3389/fphar.2021.708050

**Published:** 2021-07-22

**Authors:** Christoph Helma, Verena Schöning, Jürgen Drewe, Philipp Boss

**Affiliations:** ^1^ In Silico Toxicology Gmbh, Basel, Switzerland; ^2^ Clinical Pharmacology and Toxicology, Department of General Internal Medicine, University Hospital Bern, University of Bern, Inselspital, Bern, Switzerland; ^3^ Max Zeller Söhne AG, Romanshorn, Switzerland; ^4^ Department of Clinical Pharmacology, University Hospital Basel, University of Basel, Basel, Switzerland; ^5^ Berlin Institute for Medical Systems Biology, Max Delbrück Center for Molecular Medicine in the Helmholtz Association, Berlin, Germany

**Keywords:** mutagenicity, lazar, openbabel, CDK, machine learning, tensorflow, pyrrolizidine alkaloids

## Abstract

Random forest, support vector machine, logistic regression, neural networks and k-nearest neighbor (lazar) algorithms, were applied to a new *Salmonella* mutagenicity dataset with 8,290 unique chemical structures utilizing MolPrint2D and Chemistry Development Kit (CDK) descriptors. Crossvalidation accuracies of all investigated models ranged from 80 to 85% which is comparable with the interlaboratory variability of the *Salmonella* mutagenicity assay. Pyrrolizidine alkaloid predictions showed a clear distinction between chemical groups, where otonecines had the highest proportion of positive mutagenicity predictions and monoesters the lowest.

## 1 Introduction

The assessment of mutagenicity is an important part in the safety assessment of chemical structures, because mutations may lead to cancer and germ cells damage. The bacterial reverse mutation test (Ames test) is capable to identify substances that cause mutations (e.g., base-pair substitutions, frameshifts, insertions, deletions) and is generally used as the first step in genotoxicity and carcinogenicity assessments.

Computer based (*in silico*) mutagenicity predictions can be used in the early screening of novel compounds (e.g., drug candidates), but they are also gaining regulatory acceptance e.g. for the registration of industrial chemicals within REACH (European Chemical Agency, 2017) or the assessment of impurities in pharmaceuticals (ICH, 2017).

Currently, mutagenicity is the toxicological endpoint with the largest amount of public data for almost 10000 structures, whereas datasets for other endpoints contain typically only a few hundred compounds. The Ames test itself is relatively reproducible with an interlaboratory variability of 80–85% (Piegorsch and Zeiger, 1991).

This makes the development of mutagenicity models also interesting from a computational chemistry and machine learning point of view. The relatively large amount of public data reduces the probability of chance effects due to small sample sizes and the reliability of the underlying assay reduces the risk of overfitting experimental errors.

Within this study we attempted:

• to generate a new public mutagenicity training dataset focusing on *Salmonella typhimurium*, by combining the most comprehensive public datasets
• to compare the performance of MolPrint2D (*MP2D*) fingerprints with Chemistry Development Kit (*CDK*) descriptors for mutagenicity predictions
• to compare the performance of global QSAR models (random forests (*RF*), support vector machines (*SVM*), logistic regression (*LR*), neural nets (*NN*) with local models (lazar)

To demonstrate the application of mutagenicity models to compounds with very limited experimental data and to show their strengths and weaknesses we decided to apply them to Pyrrolizidine alkaloids (PAs).

Pyrrolizidine alkaloids (PAs) are characteristic metabolites of some plant families, mainly: Asteraceae, Boraginaceae, Fabaceae and Orchidaceae (Hartmann and Witte, 1995; Langel et al., 2011) and form a powerful defence mechanism against herbivores. PAs are heterocyclic ester alkaloids composed of a necine base (two fused five-membered rings joined by a single nitrogen atom) and a necic acid (one or two carboxylic ester arms), occurring principally in two forms, tertiary base PAs and PA N-oxides.

**FIGURE 1:**
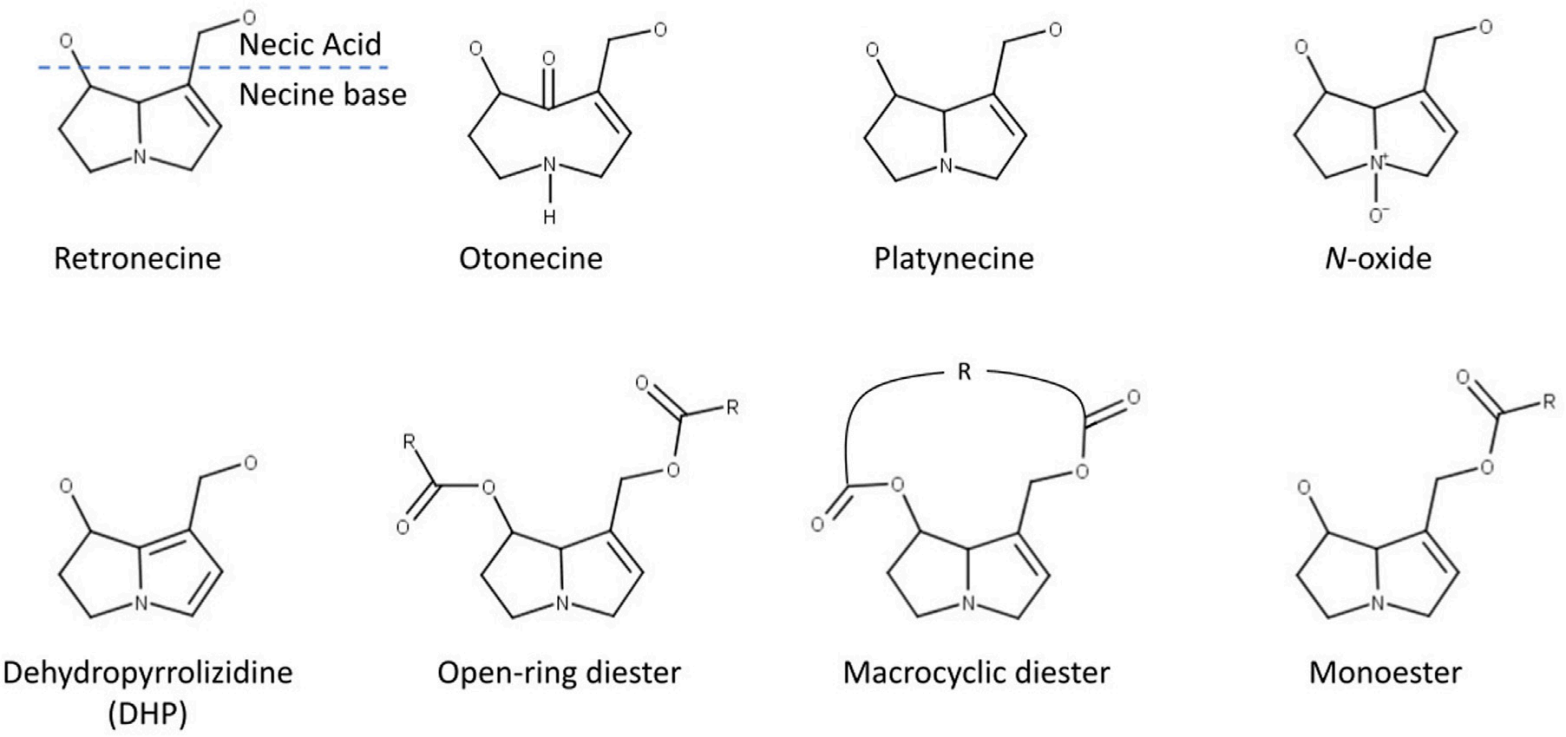
Structural features of pyrrolizidine alkaloids (modified after Schöning et al., 2017).

In mammals, PAs are mainly metabolized in the liver. There are three principal metabolic pathways for 1,2-unsaturated PAs (Chen et al.,2010):

• Detoxification by hydrolysis of the ester bond on positions C7 and C9 by non-specific esterases to release necine base and necic acid.
• N-oxidation of the necine base to form PA N-oxides, which can be either conjugated by phase II enzymes and then excreted or converted back into the corresponding parent PA (Wang et al, 2005). This detoxification pathway is not possible for otonecine-type PAs, as they are N-methylated (see Figure 1).
• Metabolic activation or toxification by oxidation (for retronecine-type PAs) or oxidative N-demethylation (for otonecine-type Pas) by cytochromes P450 isoforms CYP2B and 3A (Lin et al, 1998; Ruan et al, 2014).

The latter reactions result in the formation of dehydropyrrolizidine (DHP) that is highly reactive and causes damage by building adducts with protein, lipids and DNA (Chen et al, 2010). On the other hand, open diesters and macrocyclic PAs have a reduced detoxification due to steric hinderance of the respective esterases (Ruan et al, 2014). However, due to limited availability of pure substances, only a small number of PAs have been investigated experimentally in an Ames test. To overcome this bottleneck, the application of different machine learning models to predict mutagenic probabilities based on structures and properties could provide further insights into the mutagenicity mechanisms of PAs.

## 2 Materials and Methods

### 2.1 Data

#### 2.1.1 Mutagenicity Training Data

An identical training dataset was used for all models. The training dataset was compiled from the following sources:

• Kazius/Bursi Dataset (4,337 compounds, (Kazius et al, 2005)): http://cheminformatics.org/datasets/bursi/cas_4337.zip
• Hansen Dataset (6,513 compounds, Hansen et al, 2009)): http://doc.ml.tu-berlin.de/toxbenchmark/Mutagenicity_N6512.csv
• EFSA Dataset (695 compounds EFSA 2016)): https://data.europa.eu/euodp/data/storage/f/2017-0719T142131/GENOTOX\%20data\%20and\%20dictionary.xls

Mutagenicity classifications from Kazius and Hansen datasets were used without further processing. According to these publications, compounds were classified as mutagenic if at least one positive result has been obtained in *Salmonella typhimurium* strains TA97, TA98, TA100, TA102, TA1535, TA1537 and TA1538 either with or without metabolic activation by S9. *E. coli* results were not considered in these databases. To achieve consistency with these datasets, EFSA compounds were classified as mutagenic, if at least one positive result was found for the same *Salmonella* strains either with or without metabolic activation and as non-mutagenic if no positive result was found. The complete dataset contains chemicals from very diverse application areas (e.g., pharmaceuticals, pesticides, industrial chemicals, environmental contaminants).

Dataset merges were based on unique SMILES (*Simplified Molecular Input Line Entry Specification*, (Weininger et al, 1989)) strings of the compound structures. Duplicated experimental data with the same outcome was merged into a single value, because it is likely that it originated from the same experiment. Contradictory results were kept as multiple measurements in the database. The combined training dataset contains 8,290 unique structures and 8,309 individual measurements. Contradictory results were found for 19 substances.

Source code for all data download, extraction and merge operations is publicly available from the git repository https://git.in-silico.ch/mutagenicity-paper under a GPL3 License. The new combined dataset can be found at https://git.in-silico.ch/mutagenicity-paper/tree/mutagenicity/mutagenicity.csv.

#### 2.1.2 Pyrrolizidine Alkaloid Dataset

The pyrrolizidine alkaloid dataset was created from five independent, necine base substructure searches in PubChem (https://pubchem.ncbi.nlm.nih.gov/) and compared to the PAs listed in (EFSA, 2011) and the book by (Mattocks, 1986), to ensure, that all major PAs were included. PAs mentioned in these publications, which were not found in the downloaded substances were searched individually in PubChem and, if available, downloaded separately. Non-PA substances, duplicates, and isomers were removed from the files, but artificial PAs, even if unlikely to occur in nature, were kept. The resulting PA dataset comprised a total of 602 different PAs. Further details about the compilation of the PA dataset are described in (Schöning et al, 2017).

The PAs in the dataset were classified according to structural features. A total of nine different structural features were assigned to the necine base, to modifications of the necine base and to the necic acid (Figure 1):

For the necine base, the following structural features were chosen:

• Retronecine-type (1,2-unstaturated necine base, 392 compounds)
• Otonecine-type (1,2-unstaturated necine base, 46 compounds)
• Platynecine-type (1,2-saturated necine base, 140 compounds)

For the modifications of the necine base, the following structural features were chosen:

• N-oxide-type (84 compounds)
• Dehydropyrrolizidine-type (DHP, pyrrolic ester, 23 compounds)
• Tertiary-type (PAs which were neither from the N-oxide- nor DHP-type, 495 compounds)

For the necic acid, the following structural features were chosen:

• Monoester-type (154 compounds)
• Open-ring diester-type (163 compounds)
• Macrocyclic diester-type (255 compounds)

### 2.2 Descriptors

#### 2.2.1 MolPrint2D Fingerprints

MolPrint2D fingerprints (O’Boyle et al, 2011) use atom environments as molecular representation. They determine for each atom in a molecule, the atom types of its connected atoms to represent their chemical environment. This resembles basically the chemical concept of functional groups.

In contrast to predefined lists of fragments (e.g., FP3, FP4 or MACCs fingerprints) or descriptors (e.g., CDK) they are generated dynamically from chemical structures. This has the advantage that they can capture unknown substructures of toxicological relevance that are not included in other descriptors. In addition, they allow the efficient calculation of chemical similarities (e.g., Tanimoto indices) with simple set operations.

MolPrint2D fingerprints were calculated with the OpenBabel cheminformatics library (O’Boyle et al, 2011) for the complete training dataset with 8,290 unique structures. They can be obtained from the following locations:

Training data:

• sparse representation (https://git.in-silico.ch/mutagenicity-paper/tree/mutagenicity/mutagenicity-mp2d)
• descriptor matrix (https://git.in-silico.ch/mutagenicity-paper/tree/mutagenicity/mutagenicity-mp2d.csv.gz)

Pyrrolizidine alkaloids:

• sparse representation (https://git.in-silico.ch/mutagenicity-paper/tree/pyrrolizidine-alkaloids/pa-mp2d)
• descriptor matrix (https://git.in-silico.ch/mutagenicity-paper/tree/pyrrolizidine-alkaloids/pa-mp2d.csv)

#### 2.2.2 Chemistry Development Kit Descriptors

Molecular 1D and 2D descriptors were calculated with the PaDEL-Descriptors program (http://www.yapcwsoft.com version 2.21, (Yap, 2011)). PaDEL uses the Chemistry Development Kit (*CDK*, https://cdk.github.io/index.html) library for descriptor calculations.

As the training dataset contained 8,309 instances, it was decided to delete all instances where CDK descriptor calculations failed during pre-processing. Furthermore, 19 substances with contradictory experimental results were removed. The final training dataset contained 1,442 descriptors for 8,083 compounds.

CDK training data can be obtained from https://git.in-silico.ch/mutagenicity-paper/tree/mutagenicity/mutagenicity-cdk.csv.

The same procedure was applied for the pyrrolizidine dataset yielding descriptors for compounds. CDK features for pyrrolizidine alkaloids are available at https://git.in-silico.ch/mutagenicity-paper/tree/pyrrolizidine-alkaloids/pa-cdk.csv.

### 2.3 Algorithms

#### 2.3.1 Lazar

Lazar (*lazy structure activity relationships*) is a modular framework for read-across model development and validation. It follows the following basic workflow: For a given chemical structure lazar:

• searches in a database for similar structures (neighbors) with experimental data,
• builds a local QSAR model with these neighbors and
• uses this model to predict the unknown activity of the query compound.

This procedure resembles an automated version of read across predictions in toxicology. In machine learning terms it would be classified as a k-nearest-neighbor algorithm.

Apart from this basic workflow, lazar is completely modular and allows the researcher to use arbitrary algorithms for similarity searches and local QSAR (*Quantitative structure–activity relationship*) modeling. Algorithms used within this study are described in the following sections.

##### Feature Preprocessing

MolPrint2D features were used without preprocessing. Near zero variance and strongly correlated CDK descriptors were removed and the remaining descriptor values were centered and scaled. Preprocessing was performed with the R caret preProcess function using the methods “nzv”,“corr”,“center” and “scale” with default settings.

##### Neighbor Identification

Utilizing this modularity, similarity calculations were based both on MolPrint2D fingerprints and on CDK descriptors.

For MolPrint2D fingerprints chemical similarity between two compounds *a* and *b* is expressed as the proportion between atom environments common in both structures A∩B and the total number of atom environments A∪B (Jaccard/Tanimoto index).

$$sim=\frac{\left|A\;\cap B\right|}{\left|A\;\cup B\right|}$$ (1)

For CDK descriptors chemical similarity between two compounds *a* and *b* is expressed as the cosine similarity between the descriptor vectors *A* for *a* and *B* for *b*.

$$sim=\frac{A\cdot B}{\left|A\right|\left|B\right|}$$ (2)

Threshold selection is a trade-off between prediction accuracy (high threshold) and the number of predictable compounds (low threshold). As it is in many practical cases desirable to make predictions even in the absence of closely related neighbors, we follow a tiered approach:

• First a similarity threshold of 0.5 (MP2D/Tanimoto) or 0.9 (CDK/Cosine) is used to collect neighbors, to create a local QSAR model and to make a prediction for the query compound. This are predictions with high confidence.
• If any of these steps fails, the procedure is repeated with a similarity threshold of 0.2 (MP2D/Tanimoto) or 0.7 (CDK/Cosine) and the prediction is flagged with a warning that it might be out of the applicability domain of the training data (low confidence).
• These similarity thresholds are the default values chosen by software developers and remained unchanged during the course of these experiments.

Compounds with the same structure as the query structure are automatically eliminated from neighbors to obtain unbiased predictions in the presence of duplicates.

##### Local QSAR Models and Predictions

Only similar compounds (neighbors) above the threshold are used for local QSAR models. In this investigation, we are using a weighted majority vote from the neighbor’s experimental data for mutagenicity classifications. Probabilities for both classes (mutagenic/non-mutagenic) are calculated according to the following formula and the class with the higher probability is used as prediction outcome.

$$p_{c}=\frac{\sum {\text{sim}}_{\mathrm{n,c}}}{\sum {\text{sim}}_{n}}$$ (3)

$p_{c}$ Probability of class c (e.g. mutagenic or non-mutagenic) ${\sum \mathrm{si}m_{\mathrm{n,c}}}^{\text{​}}$ Sum of similarities of neighbors with class c ${{\sum \text{sim}}_{n}}^{\text{​}}$ Sum of all neighbors.

##### Applicability Domain

The applicability domain (AD) of lazar models is determined by the structural diversity of the training data. If no similar compounds are found in the training data no predictions will be generated. Warnings are issued if the similarity threshold had to be lowered from 0.5 to 0.2 in order to enable predictions. Predictions without warnings can be considered as close to the applicability domain (*high confidence*) and predictions with warnings as more distant from the applicability domain (*low confidence*). Quantitative applicability domain information can be obtained from the similarities of individual neighbors.

##### Validation

10-fold cross validation was performed for model evaluation.

##### Pyrrolizidine Alkaloid Predictions

For the prediction of pyrrolizidine alkaloids models were generated with the MP2D and CDK training datasets. The complete feature set was used for MP2D predictions, for CDK predictions the intersection between training and pyrrolizidine alkaloid features was used.

##### Availability

• Source code for this manuscript (GPL3): https://git.in-silico.ch/lazar/tree/?h=mutagenicity-paper
• Crossvalidation experiments (GPL3): https://git.in-silico.ch/lazar/tree/models/?h=mutagenicity-paper
• Pyrrolizidine alkaloid predictions (GPL3): https://git.in-silico.ch/lazar/tree/predictions/?h=mutagenicity-paper
• Public web interface: https://lazar.in-silico.ch

#### 2.3.2 Tensorflow Models

##### Feature Preprocessing

For preprocessing of the CDK features we used a quantile transformation to a uniform distribution. MP2D features were not preprocessed.

##### Random Forests (*RF*)

For the random forest classifier we used the parameters n_estimators = 1,000 and max_leaf_nodes = 200. For the other parameters we used the scikit-learn default values.

##### Logistic Regression (SGD) (*LR-Ssgd*)

For the logistic regression we used a combination of five trained models. For each model we used a batch size of 64 and trained for 50 epochs. As an optimizer ADAM was chosen. For the other parameters we used the tensorflow default values.

##### Logistic Regression (Scikit) (*LR-Scikit*)

For the logistic regression we used as parameters the scikit-learn default values.

##### Neural Nets

For the neural network we used a combination of five trained models. For each model we used a batch size of 64 and trained for 50 epochs. As an optimizer ADAM was chosen. The neural network had four hidden layers with 64 nodes each and a ReLu activation function. For the other parameters we used the tensorflow default values.

##### Support Vector Machines

We used the SVM implemented in scikit-learn. We used the parameters kernel = “rbf,” gamma = “scale”. For the other parameters we used the scikit-learn default values.

##### Validation

10-fold cross-validation was used for all Tensorflow models.

##### Pyrrolizidine Alkaloid Predictions

For the prediction of pyrrolizidine alkaloids we trained the model described above on the training data. For training and prediction only the features were used that were in the intersection of features from the training data and the pyrrolizidine alkaloids.

##### Availability

Jupyter notebooks for these experiments can be found at the following locations.

##### Crossvalidation:

• MolPrint2D fingerprints: https://git.in-silico.ch/mutagenicity-paper/tree/crossvalidations/tensorflow/prediction-v5-norm.ipynb
• CDK descriptors: https://git.in-silico.ch/mutagenicity-paper/tree/crossvalidations/tensorflow/prediction-v5-ext.ipynb

##### Pyrrolizidine Alkaloids:

• MolPrint2D fingerprints: https://git.in-silico.ch/mutagenicity-paper/tree/pyrrolizidine-alkaloids/tensorflow/prediction-v5-ext-ext-Padel-2D.ipynb
• CDK descriptors: https://git.in-silico.ch/mutagenicity-paper/tree/pyrrolizidine-alkaloids/tensorflow/prediction-v5-ext-Padel-2D.ipynb

## 3 Results

### 3.1 10-Fold Crossvalidations

Crossvalidation results are summarized in the following tables: Table 1 shows results with MolPrint2D descriptors and Table 2 with CDK descriptors.

**TABLE 1:** Summary of crossvalidation results with MolPrint descriptors (lazar-HC, lazar with high confidence, lazar-all, all lazar predictions, RF, random forests, LR-sgd, logistic regression (stochastic gradient descent), LR-scikit, logistic regression (scikit), NN, neural networks, SVM, support vector machines).

	Lazar-HC	Lazar-all	RF	LR-sgd	LR-scikit	NN	SVM
Accuracy	84	82	80	84	84	84	84
True positive rate	89	85	78	83	83	82	83
True negative rate	78	78	82	84	85	85	86
Positive predictive value	83	80	81	84	84	84	85
Negative predictive value	86	84	80	84	84	83	84
Nr. predictions	5,864	7,782	8,303	8,303	8,303	8,303	8,303

**TABLE 2:** Summary of crossvalidation results with CDK descriptors (lazar-HC, lazar with high confidence, lazar-all: all lazar predictions, RF, random forests, LR-sgd, logistic regression (stochastic gradient descent), LR-scikit, logistic regression (scikit), NN, neural networks, SVM, support vector machines).

	Lazar-HC	Lazar-all	RF	LR-sgd	LR-scikit	NN	SVM
Accuracy	85	82	84	79	80	85	82
True positive rate	87	84	81	81	80	85	82
True negative rate	82	80	86	78	80	85	82
Positive predictive value	85	81	85	79	80	85	82
Negative predictive value	85	82	82	80	80	85	82
Nr. predictions	4,872	7,353	8,077	8,077	8,077	8,077	8,077

**FIGURE 2:**
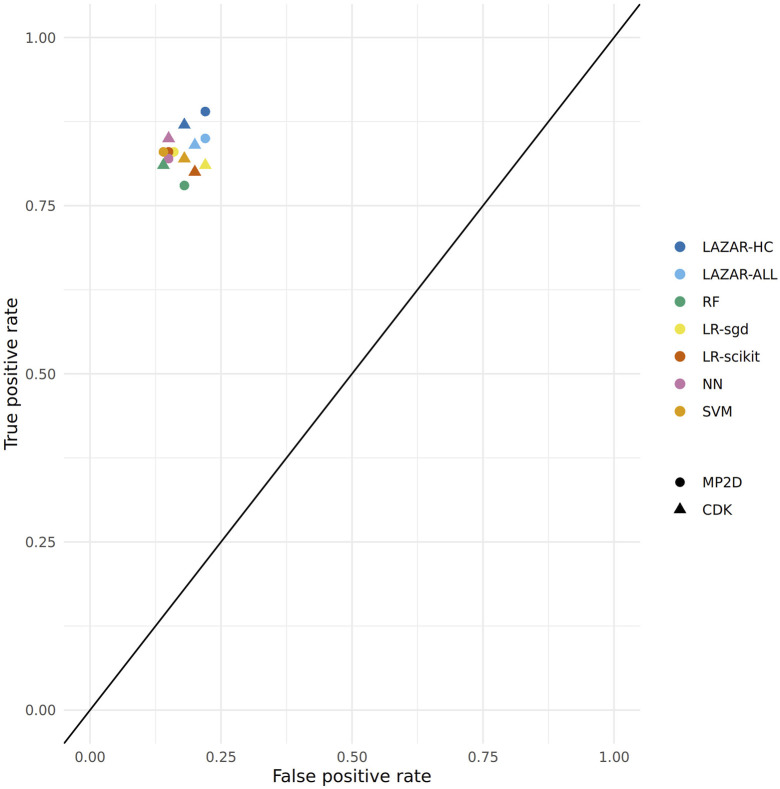
ROC plot of crossvalidation results (lazar-HC, lazar with high confidence, lazar-all: all lazar predictions, RF, random forests, LR-sgd, logistic regression (stochastic gradient descent), LR-scikit, logistic regression (scikit), NN, neural networks, SVM, support vector machines).

Figure 2 depicts the position of all crossvalidation results in receiver operating characteristic (ROC) space.

Confusion matrices for all models are available from the git repository https://git.in-silico.ch/mutagenicity-paper/tree/crossvalidations/confusion-matrices/, individual predictions can be found in https://git.in-silico.ch/mutagenicity-paper/tree/crossvalidations/predictions/.

All investigated algorithm/descriptor combinations give accuracies between (80 and 85%) which is equivalent to the experimental variability of the *Salmonella typhimurium* mutagenicity bioassay (80–85%, Piegorsch and Zeiger, 1991)). Sensitivities and specificities are balanced in all of these models.

### 3.2 Pyrrolizidine Alkaloid Mutagenicity Predictions

Mutagenicity predictions of 602 pyrrolizidine alkaloids (PAs) from all investigated models can be downloaded from https://git.in-silico.ch/mutagenicity-paper/tree/pyrrolizidine-alkaloids/pa-predictions.csv. A visual representation of all PA predictions can be found at https://git.in-silico.ch/mutagenicity-paper/tree/pyrrolizidine-alkaloids/pa-predictions.pdf.

For the visualization of the position of pyrrolizidine alkaloids in respect to the training data set we have applied t-distributed stochastic neighbor embedding (t-SNE, (Maaten and Hinton, 2008) for MolPrint2D and CDK descriptors. t-SNE maps each high-dimensional object (chemical) to a two-dimensional point, maintaining the high-dimensional distances of the objects. Similar objects are represented by nearby points and dissimilar objects are represented by distant points. t-SNE coordinates were calculated with the R Rtsne package using the default settings (perplexity = 30, theta = 0.5, max_iter = 1,000).

**FIGURE 3:**
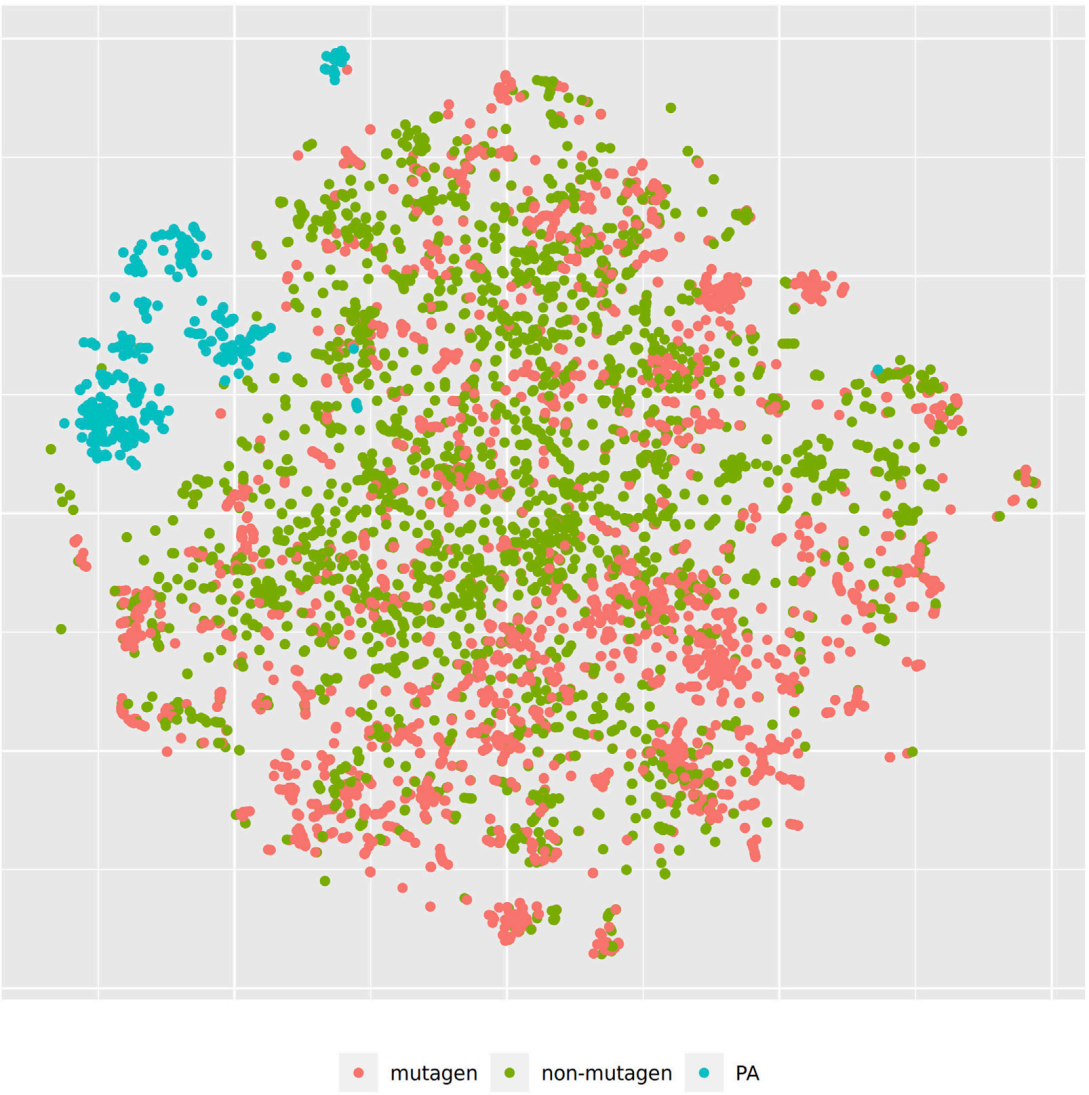
t-SNE visualization of mutagenicity training data and pyrrolizidine alkaloids (PA) in MP2D space.

Figure 3 shows the t-SNE of pyrrolizidine alkaloids (PA) and the mutagenicity training data in MP2D space (Tanimoto/Jaccard similarity), which resembles basically the structural diversity of the investigated compounds.

**FIGURE 4:**
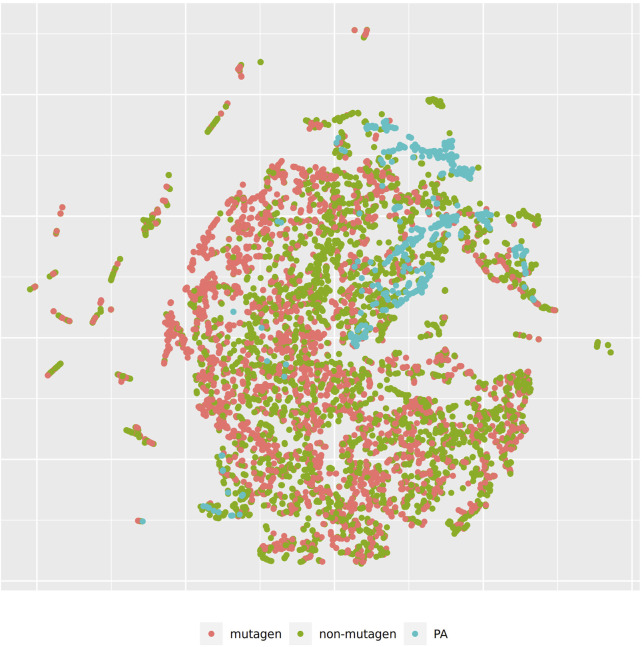
t-SNE visualization of mutagenicity training data and pyrrolizidine alkaloids (PA) in CDK space.

Figure 4 shows the t-SNE of pyrrolizidine alkaloids (PA) and the mutagenicity training data in CDK space (Euclidean similarity), which resembles basically the physical-chemical properties of the investigated compounds.

**FIGURE 5:**
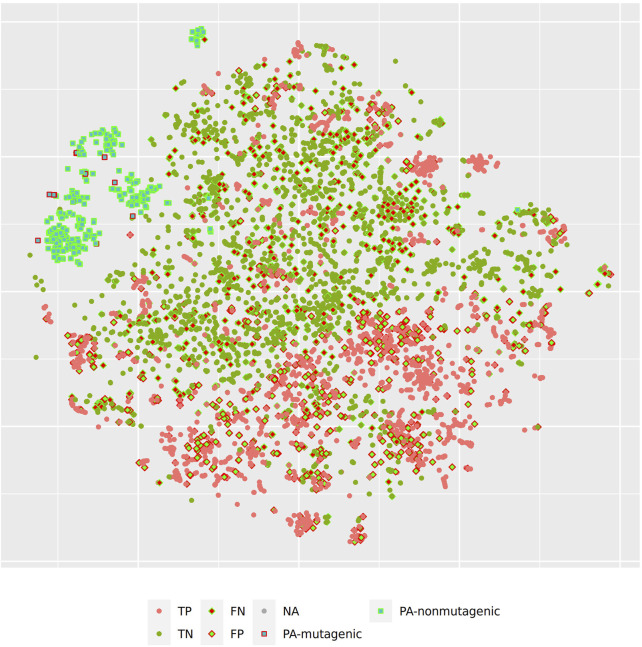
t-SNE visualization of MP2D random forest predictions.

**FIGURE 6:**
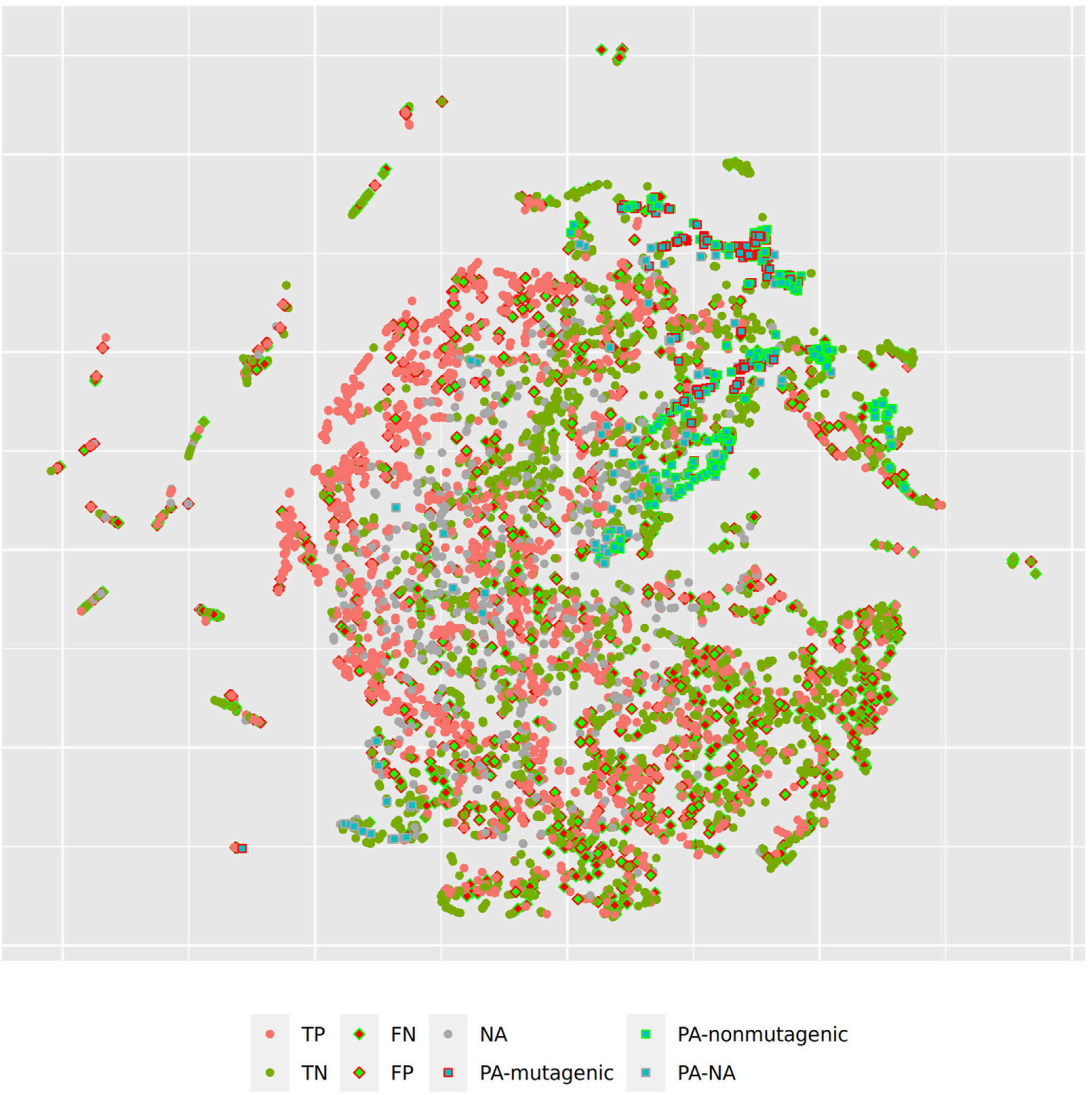
t-SNE visualization of all CDK lazar predictions.

Figure 5 and Figure 6 depict two example pyrrolizidine alkaloid mutagenicity predictions in the context of training data. t-SNE visualisations of all investigated models can be downloaded from https://git.in-silico.ch/mutagenicity-paper/figures.

Table 3 summarizses the outcome of pyrrolizidine alkaloid predictions from all models with MolPrint2D and CDK descriptors.

**TABLE 3:** Summary of pyrrolizidine alkaloid predictions.

Model	MP2D mutagenic	Nr. predictions	CDK mutagenic	Nr. predictions
Lazar-all	20% (111)	93% (560)	39% (193)	83% (500)
Lazar-HC	25% (76)	50% (301)	45% (111)	41% (246)
RF	5% (28)	100% (602)	2% (10)	100% (602)
LR-sgd	21% (127)	100% (602)	16% (97)	100% (602)
LR-scikit	20% (118)	100% (602)	15% (88)	100% (602)
NN	21% (124)	100% (602)	25% (150)	100% (602)
SVM	14% (82)	100% (602)	3% (19)	100% (602)

**FIGURE 7:**
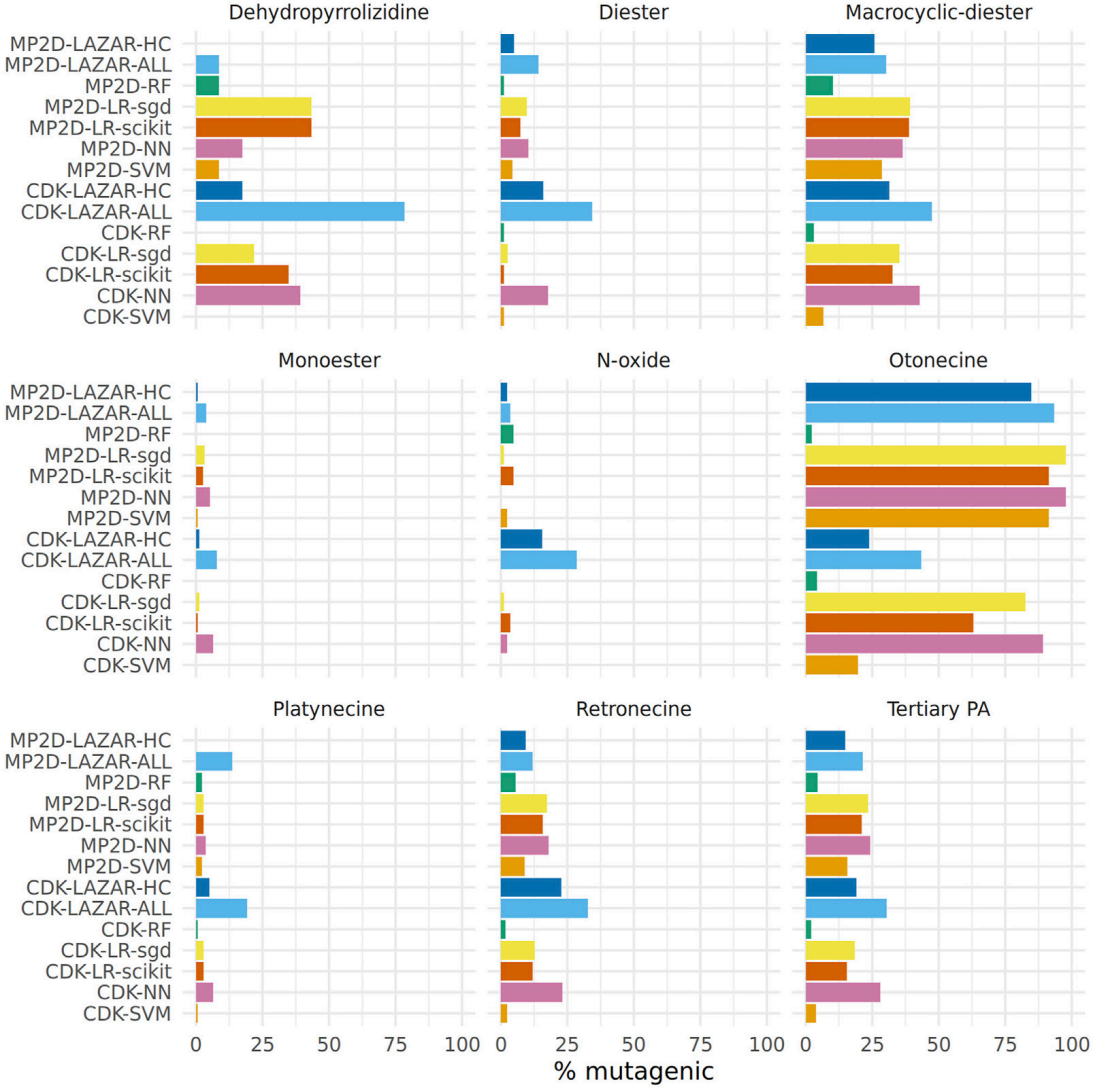
Summary of pyrrolizidine alkaloid predictions.

Figure 7 displays the proportion of positive mutagenicity predictions from all models for the different pyrrolizidine alkaloid groups. Tensorflow models predicted all 602 pyrrolizidine alkaloids, lazar MP2D models predicted 560 compounds (301 with high confidence) and lazar CDK models 500 compounds (246 with high confidence).

For the lazar-HC model, only 50/41% of the PA dataset were within the stricter similarity thresholds of 0.5/0.9 (MP2D/CDK). Reduction of the similarity threshold to 0.2/0.5 in the lazar-all model increased the amount of predictable PAs to 93/83%. As the other ML models do not consider applicability domains, all PAs were predicted.

Although most of the models show similar accuracies, sensitivities and specificities in crossvalidation experiments some of the models (MPD-RF, CDK-RF and CDK-SVM) predict a lower number of mutagens (2–5%) than the majority of the models (14–25%, Table 3, Figure 7).

Over all models, the mean value of mutagenic predicted PAs was highest for otonecines (65%, 407/623), followed by macrocyclic diesters (31%, 1,042/3,356), dehydropyrrolizidines (27%, 74/268), tertiary PAs (19%, 1,201/6,307) and retronecines (15%, 762/5,054).

When excluding the aforementioned three deviating models, the rank order stays the same, but the percentage of mutagenic PAs is higher.

The following rank order for mutagenic probability can be deduced from the results of all models taken together:

Necine base: Platynecine < Retronecine << Otonecine.

Necic acid: Monoester < Diester << Macrocyclic diester.

Modification of necine base: N-oxide < Tertiary PA < Dehydropyrrolizidine.

## 4 Discussion

### 4.1 Data

A new training dataset for *Salmonella* mutagenicity was created from three different sources (Kazius et al, 2005; Hansen et al, 2009); EFSA, 2016). It contains 8,290 unique chemical structures, which is according to our knowledge the largest public mutagenicity dataset presently available. The new training data can be downloaded from https://git.in-silico.ch/mutagenicity-paper/tree/mutagenicity/mutagenicity.csv.

### 4.2 Algorithms

Lazar is formally a *k-nearest-neighbor* algorithm that searches for similar structures for a given compound and calculates the prediction based on the experimental data for these structures. The QSAR literature calls such models frequently *local models*, because models are generated specifically for each query compound. The investigated tensorflow models are in contrast *global models*, i.e. a single model is used to make predictions for all compounds. It has been postulated in the past, that local models are more accurate, because they can account better for mechanisms that affect only a subset of the training data.

Table 1, Table 2 and Figure 2 show that the crossvalidation accuracies of all models are comparable to the experimental variability of the *Salmonella typhimurium* mutagenicity bioassay (80–85% according to (Piegorsch and Zeiger, 1991). All of these models have balanced sensitivity (true positive rate) and specificity (true negative rate) and provide highly significant concordance with experimental data (as determined by McNemar’s Test). This is a clear indication that *in silico* predictions can be as reliable as the bioassays. Given that the variability of experimental data is similar to model variability it is impossible to decide which model gives the most accurate predictions, as models with higher accuracies might just approximate experimental errors better than more robust models.

Our results do not support the assumption that local models are superior to global models for classification purposes. For regression models (lowest observed effect level) we have found however that local models may outperform global models (Helma et al, 2018) with accuracies similar to experimental variability.

**FIGURE 8:**
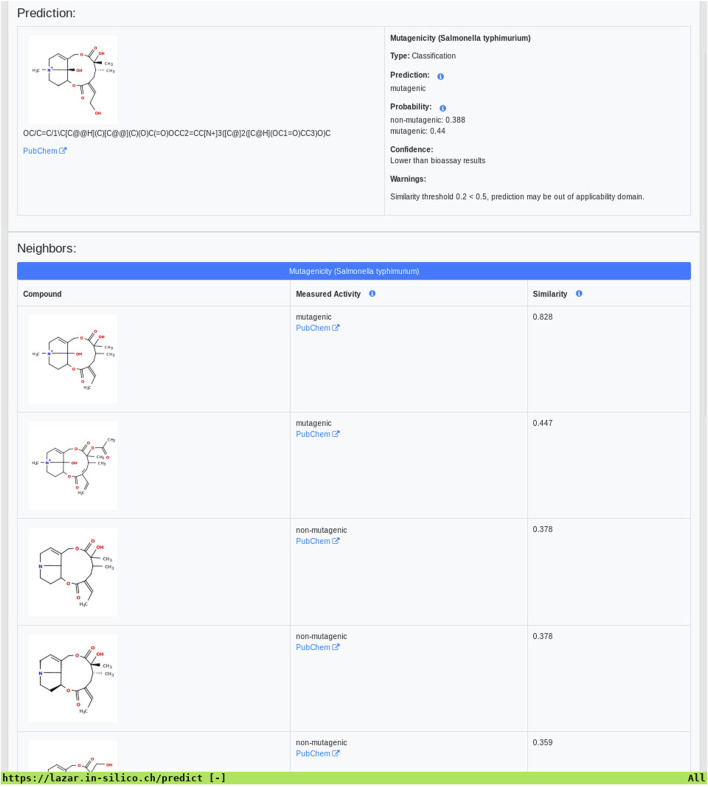
Lazar screenshot of 12,21-Dihydroxy-4-methyl-4,8-secosenecinonan-8,11,16-trione mutagenicity prediction.

As all investigated algorithms give similar accuracies the selection will depend more on practical considerations than on intrinsic properties. Nearest neighbor algorithms like lazar have the practical advantage that the rationales for individual predictions can be presented in a straightforward manner that is understandable without a background in statistics or machine learning (a screenshot of the mutagenicity prediction for 12,21-Dihydroxy-4-methyl-4,8-secosenecinonan-8,11,16-trione is depicted in Figure 8). This allows a critical examination of individual predictions and prevents blind trust in models that are intransparent to users with a toxicological background.

### 4.3 Descriptors

This study uses two types of descriptors for the characterization of chemical structures:

*MolPrint2D* fingerprints (MP2D, (Bender et al, 2004) use atom environments (i.e., connected atom types for all atoms in a molecule) as molecular representation, which resembles basically the chemical concept of functional groups. MP2D descriptors are used to determine chemical similarities in the default lazar settings, and previous experiments have shown, that they give more accurate results than predefined fingerprints (e.g., MACCS, FP2-4).

*Chemistry Development Kit* (CDK, Willighagen et al., 2017) descriptors were calculated with the PaDEL graphical interface (Yap, 2011). They include 1D and 2D topological descriptors as well as physical-chemical properties.

All investigated algorithms obtained models within the experimental variability for both types of descriptors (Table 1, Table 2, Figure 2).

Given that similar predictive accuracies are obtainable from both types of descriptors the choice depends once more on practical considerations:

MolPrint2D fragments can be calculated very efficiently for every well defined chemical structure with OpenBabel (O’Boyle et al, 2011). CDK descriptor calculations are in contrast much more resource intensive and may fail for a significant number of compounds (from 8,290).

MolPrint2D fragments are generated dynamically from chemical structures and can be used to determine if a compound contains structural features that are absent in training data. This feature can be used to determine applicability domains. CDK descriptors contain in contrast a predefined set of descriptors with unknown toxicological relevance.

MolPrint2D fingerprints can be represented very efficiently as sets of features that are present in a given compound which makes similarity calculations very efficient. Due to the large number of substructures present in training compounds, they lead however to large and sparsely populated datasets, if they have to be expanded to a binary matrix (e.g., as input for tensorflow models). CDK descriptors contain in contrast in every case matrices with 1,442 columns which can cause substantial computational overhead.

### 4.4 Pyrrolizidine Alkaloid Mutagenicity Predictions

#### 4.4.1 Algorithms and Descriptors

Figure 7 shows a clear differentiation between the different pyrrolizidine alkaloid groups. Nevertheless differences between predictions from different algorithms and descriptors (Table 3) were not expected based on crossvalidation results.

In order to investigate, if any of the investigated models show systematic errors in the vicinity of pyrrolizidine-alkaloids we have performed a detailled t-SNE analysis of all models (see Figure 5 and Figure 6 for two examples, all visualisations can be found at https://git.in-silico.ch/mutagenicity-paper/tree/figures).

None of the models showed obvious deviations from their expected behavior, so the reason for the disagreement between some of the models remains unclear at the moment. It is however possible that some systematic errors are covered up by converting high dimensional spaces to two coordinates and are thus invisible in t-SNE visualisations.

Only two compounds from the PA dataset (Senecivernine and Retronecine) are part of the training set. Both are non-mutagenic and were predicted as non-mutagenic by all models (instances have been removed from the training set for unbiased predictions). Despite the exact concordance, we cannot draw any general conclusions about model performance based on two examples with a single outcome.

#### 4.4.2 Necic Acid

The rank order of the necic acid is comparable in all models. PAs from the monoester type had the lowest genotoxic probability, followed by PAs from the open-ring diester type. PAs with macrocyclic diesters had the highest genotoxic probability. The result fits well with current state of knowledge: in general, PAs, which have a macrocyclic diesters as necic acid, are considered to be more mutagenic than those with an open-ring diester or monoester (EFSA, 2011; Fu et al, 2004). As pointed out above, open diesters and macrocyclic PAs have a reduced detoxification due to steric hinderance of the respective esterases (Ruan et al, 2014). This was also confirmed by more recent studies, confirming that macrocyclic- and open-diesters are more genotoxic *in vitro* than monoesters (Allemang et al, 2018; Louisse et al, 2019; Hadi et al, 2021).

#### 4.4.3 Necine Base

In the rank order of necine base PAs, platynecine is the least mutagenic, followed by retronecine, and otonecine. Saturated PAs of the platynecine-type are generally accepted to be less or non-mutagenic and have been shown in *in vitro* experiments to form no DNA-adducts (Xia et al, 2013). In literature, otonecine-type PAs were shown to be more mutagenic than those of the retronecine-type (Li et al, 2013).

#### 4.4.4 Modifications of Necine Base

The group-specific results reflect the expected relationship between the groups: the low mutagenic probability of *N*-oxides and the high probability of dehydropyrrolizidines (DHP) (Chen et al., 2010). However, *N*-oxides may be *in vivo* converted back to their parent mutagenic/tumorigenic parent PA (Yan et al, 2008), on the other hand they are highly water soluble and generally considered as detoxification products, which are *in vivo* quickly renally eliminated (Chen et al., 2010).

DHP are regarded as the toxic principle in the metabolism of PAs, and are known to produce protein- and DNA-adducts (Chen et al., 2010). None of our investigated models did meet this expectation and all of them predicted the majority of DHP as non-mutagenic. However, the following issues need to be considered: On the one hand, all DHP were outside of the stricter applicability domain of MP2D lazar. This indicates that they are structurally very different than the training data and might be out of the applicability domain of all models based on this training set. In addition, DHP has two unsaturated double bounds in its necine base, making it highly reactive. DHP and other comparable molecules have a very short lifespan *in vivo*, and usually cannot be used in *in vitro* experiments.

Overall the low number of positive mutagenicity predictions was unexpected. PAs are generally considered to be genotoxic, and the mode of action is also known. Therefore, the fact that some models predict the majority of PAs as not mutagenic seems contradictory. To understand this result, the experimental basis of the training dataset has to be considered. The training dataset is based on the *Salmonella typhimurium* mutagenicity bioassay (Ames test). There are some studies, which show mutagenicity of PAs in the Ames test (Chen et al., 2010). Also, Rubiolo et al. (1992) examined several different PAs and several different extracts of PA-containing plants in the Ames test. They found that the Ames test was indeed able to detect mutagenicity of PAs, but in general, appeared to have a low sensitivity. The pre-incubation phase for metabolic activation of PAs by microsomal enzymes was the sensitivity-limiting step. This could very well mean that the low sensitivity of the Ames test for PAs is also reflected in the investigated models.

In summary, we found marked differences in the predicted genotoxic probability between the PA groups: most mutagenic appeared the otonecines and macrocyclic diesters, least mutagenic the platynecines and the mono- and diesters. These results are comparable with *in vitro* measurements in hepatic HepaRG cells (Louisse et al, 2019), where relative potencies (RP) were determined: for otonecines and cyclic diesters RP = 1, for open diesters RP = 0.1 and for monoesters RP = 0.01.

Due to a lack of differential data, European authorities based their risk assessment in a worst-case approach on lasiocarpine, for which sufficient data on genotoxicity and carcinogenicity were available (HMPC, 2014; EMA, 2020). Our data further support a tiered risk assessment based on *in silico* and experimental data on the relative potency of individual PAs as already suggested by other authors (Merz and Schrenk, 2016; Louisse et al, 2019; Rutz et al, 2020).

The practical question how to choose model predictions in the absence of experimental data remains open. Tensorflow predictions do not include applicability domain estimations and the rationales for predictions cannot be traced by toxicologists. Transparent models like lazar may have an advantage in this context, because they present rationales for predictions (similar compounds with experimental data) which can be accepted or rejected by toxicologists and provide validated applicability domain estimations.

## 5 Conclusion

A new public *Salmonella* mutagenicity training dataset with 8,309 experimental results was created and used to train lazar and Tensorflow models with MolPrint2D and CDK descriptors. All investigated algorithm and descriptor combinations showed accuracies comparable to the interlaboratory variability of the Ames test.

Pyrrolizidine alkaloid predictions showed a clear separation between different classes of PAs which were generally in accordance with the current toxicological knowledge about these compounds. Some of the models showed however a substantially lower number of mutagenicity predictions, despite similar crossvalidation results and we were unable to identify the reasons for this discrepancy within this investigation.

Our data show that large difference exist with regard to mutagenic probabilities between different pyrrolizidine subgroups. To adjust risk assessment of pyrrolizidine contamination, our data supports a tiered risk assessment based on *in silico* predictions and experimental data of individual pyrrolizidine alkaloids.

## Data Availability

Data, source code, results and supplementary material presented in this study is publicly available at the Git repository https://git.in-silico.ch/mutagenicity-paper. Further inquiries can be directed to the corresponding authors.
